# Powered mobility device use in residential aged care: a retrospective audit of incidents and injuries

**DOI:** 10.1186/s12877-023-04073-z

**Published:** 2023-06-11

**Authors:** Natalie C. Dickson, Apeksha R. Gohil, Carolyn A. Unsworth

**Affiliations:** 1 Institute of Health and Wellbeing, Federation University, Gippsland Campus, PO Box 3191, Churchill, VIC 3841 Australia; 2 College of Healthcare Sciences, James Cook University, Townsville, QLD Australia; 3 Department of Neurosciences, Monash University, Clayton, VIC Australia; 4 Department of Occupational Therapy, Jönköping University, Jönköping, Sweden

**Keywords:** Powered wheelchair, Mobility scooter, Incidents, Injuries

## Abstract

**Background:**

Powered wheelchairs and motorised mobility scooters, collectively called powered mobility devices (PMD), are highly valued by older Australians, including those living in residential care, to facilitate personal and community mobility. The number of PMDs in residential aged care is expected to grow proportionally with that of the wider community, however, there is very little literature on supporting residents to use PMDs safely. Prior to developing such supports, it is important to understand the frequency and nature of any incidents experienced by residents whilst using a PMD. The aim of this study was to determine the number and characteristics of PMD use related incidents occurring in a group of residential aged care facilities in a single year in one state in Australia including incident type, severity, assessment, or training received and outcomes on follow-up for PMD users living in residential aged care.

**Methods:**

Analysis of secondary data, including documentation of PMD incidents and injuries for one aged care provider group over 12 months retrospectively. Follow-up data were gathered 9–12 months post incident to review and record the outcome for each PMD user.

**Results:**

No fatalities were recorded as a direct result of PMD use and 55 incidents, including collisions, tips, and falls, were attributed to 30 residents. Examination of demographics and incident characteristics found that 67% of residents who had incurred incidents were male, 67% were over 80 years of age, 97% had multiple diagnoses and 53% had not received training to use a PMD. Results from this study were extrapolated to project that 4,453 PMD use related incidents occur every year within Australian residential aged care facilities, with the potential for outcomes such as extended recovery, fatality, litigation, or loss of income.

**Conclusion:**

This is the first time that detailed incident data on PMD use in residential aged care has been reviewed in an Australian context. Illuminating both the benefits and the potential risks of PMD use emphasizes the need to develop and improve support structures to promote safe PMD use in residential aged care.

Powered mobility devices (PMD), such as mobility scooters or powered wheelchairs, are highly valued by older Australians to supplement or restore personal and community mobility for everyday tasks [1, 2]. A PMD can bridge the gap of lost mobility independence, renew the ability to participate in valued roles and activities, and contribute to autonomy, self-esteem, and personal well-being [3–7]. PMD use in the community is growing proportionally with the increasing number of people aged over 65 who need support with mobility [8–11]. The prevalence of PMD use in Australia in 2012 was estimated in a survey which indicated there were 231,000 mobility scooter users in the adult population (age 18 and over). Of this number, 49% were over 60 years, therefore, there were approximately 113,000 older adult mobility scooter users (not including powered wheelchairs) [12]. In 2012 in Canada 0.3% of the population or approximately 45,273 [10] and in North America in 2002 approximately 122,000 [13] community-living older adults were using powered wheelchairs or mobility scooters. Similarly, PMDs are used in residential aged care facilities and this number continues to grow as residents become more familiar with this technology [14]. Residential aged care in Australia, by definition, provides a range of accommodation with catering, 24-h personal care and domestic services, nursing, and general health care services, for older people who are unable to continue living independently in their own homes [15]. Other terms used to describe these facilities may include, care homes, long term care, nursing homes or skilled nursing facilities. The providers of aged care accommodation in Australia are required to follow quality standards for accreditation, one of which is to ensure the choices of each resident are accommodated, including activities that carry some risk [15].

With an increasing number of residents enjoying the freedoms associated with PMD use, the corresponding potential risk for incidents and injuries also increases [9, 14, 16–19]. An investigation into serious injury data for mobility scooter users (excluding powered wheelchairs) aged over 60 years in 2011, found 442 injuries due to falls from ‘unspecified pedestrian conveyances’ presented to hospitals across Australia between 2006–2008 and an increase in hospital presentations in the state of Victoria of 255% in the same period [20]. More recently, it was reported that 2,477 Australians over 60 years old were hospitalised between 2011 and 2016, which showed an increase of more than twice the annual average [21]. However, multiple authors suggested that these injury data sets were under-estimated due to limitations in the data coding system [18, 20, 21]. Beyond injury, the 2011 report recorded 62 fatalities related to the use of mobility scooters nationally between 2000–2010 and a further study concurred with similar numbers of seven to eight mobility scooter fatalities in Australia every year [18, 20]. These studies showed that six out of ten mobility scooter related fatalities were attributed to people in the 80–89 age range, however, it was unclear from these data how many of the incidents were sustained by people living in residential aged care facilities [18, 20, 21].

Although there is a well-established literature on PMD use and incidents in community settings [22, 23], there is limited evidence of PMD related incidents in the residential aged care context [24]. The available incident data for older Australians living in residential aged care facilities suggests that between 2006–2016, there were approximately 17 mobility scooter related fatalities over a ten-year period, although it is difficult to retrospectively estimate the number of scooter users in Australian residential care facilities at that time [21]. This equates to one to two PMD use related fatalities in Australian residential aged care facilities every year. The Australian Institute of Health and Welfare [21] sourced these data from a detailed search of the National Coronial Information System for the ten-year period and only included incidents coded to ‘motorised mobility scooter’, ‘motorised wheelchair’, ‘powered scooter’, ‘buggy’, or ‘gopher’.

The terminology used to differentiate PMD types, and for coding, was noted to vary considerably in the literature. However, PMDs are easily divisible into two types, the first a motorised mobility scooter with three or four wheels, a seat on a platform, and a central steering column, also known as a tiller. The second, a powered wheelchair with four or six wheels, plus a seat or frame for specialised seating, usually controlled with a joystick mounted to an armrest [2, 22, 25]. The motorised mobility scooter generally has a larger footprint and turning circle plus side access and provides minimal postural support, most suited to supplementing outdoor mobility for people with independent transfers, on and off the device [26]. A powered wheelchair is more suited to people needing greater mobility support, or people who have no independent mobility, with a smaller footprint and turning circle, front access for assisted transfers, flexible options for control and postural support, plus suspension for varied terrain [22, 26]. Both devices can be used by older adults who live in residential aged care facilities, however, it is important to differentiate between them because the devices are not equal in function nor features, which is why prescription based on individual assessment is recommended, as seen in the World Health Organisation’s eight key steps for wheelchair service delivery [27].

Although considered important for safe use, there is no mandatory PMD assessment or training in any state of Australia, unless required by a funding body, and there is no requirement to register PMD ownership with a governing body except in Queensland [2, 25, 28, 29]. Furthermore, no international literature could be located that specified contemporary, procedural support to apply to PMD use in residential aged care settings. In the absence of procedural support and where staff are unqualified to investigate incidents, modify equipment, or provide skills re-training, immediate removal of a PMD is a common outcome after an incident [30, 31]. Removal of a PMD, whether warranted or not, can cause psychological harm to a PMD user whose ‘wheels’ have become integrated with self-identity over time, akin to ‘legs’ [13, 32, 33]. This presents a challenge which requires a method for balancing provision of autonomy for residents using a PMD, with safety for all stakeholders within the environment, including other residents, visitors, and staff [14]. A coordinated approach to support the ongoing use of PMDs within residential aged care services is needed equally to that of the general community [27]. To further investigate the balance between PMD autonomy and safety, it was identified that a greater understanding of PMD incidents within the residential aged care context was needed. This study aimed to investigate the number and characteristics of PMD use related incidents in residential aged care, to inform subsequent studies and progress this area of need.

## Methods

### Design

Secondary data analysis was undertaken following a retrospective audit that involved searching and extracting data from a pre-existing data set pertaining to PMD use related incidents in residential aged care settings. The study used both quantitative notes and simple descriptive data in a pragmatist manner [34–36] to develop a detailed collection of incidents and injuries which provides evidence to support future research on safe PMD use.

### Data collection

In this study, data from individual incident reports and progress notes related to PMD use, were collected from Platinum 5 [37], a software system used within an aged care provider organisation in the state of New South Wales, Australia to manage resident data. Platinum 5 was designed for the aged care sector, providing a system to manage all aspects of care delivery, quality, and compliance [37]. This software system can include the standardised assessments that an organisation wants to include for residents, such as the Psychogeriatric Assessment Scale (PAS) [38]. The provider group owned and managed 33 facilities at the time of this study, with an average of 65 beds per facility, turning over at an unspecified rate each year, housing an estimated 2,210 people. All facilities were compliant with the Aged Care Quality Standards and accessibility features included ramp access, lifts, and wide doorways and hallways [15]. Data were collected for incidents occurring over the period 01/01/2018 – 31/12/2018, with follow-up data collected nine to 12 months later, up to 31/12/2019.

Incident reports concerning PMD use in Platinum 5 were identified using the key search terms; ‘electric wheelchair’, ‘power* wheelchair’, ‘electric’, ‘scooter’, ‘gopher’, ‘buggy’, ‘motorised’ and ‘motorized’. Incidents were identified one facility at a time by search function and then hand sorted to ensure that PMD use was indicated. Where present, assessment and training data were gathered from allied health progress notes, through hand searching a date range around the incident/s using the search terms, ‘occupation*’, ‘physio*’, ‘allied health’, ‘assessment’, ‘training’, in addition to the terms searched for PMDs noted above. A data extraction table was developed to organise and record the data collected, including resident demographic details, PMD type, PMD incident severity, assessment or training details, individual functional status, cognitive screen result, and any related notes. The International Statistical Classification of Diseases and Related Health Problems (ICD-11) was used to code each diagnosis listed for each resident [39].

The information collected for each resident was organised into a simple count of incidents, and demographic information as follows; number of incidents; incident type; PMD type; resident demographic information including, age, sex, diagnosis – type and number, incident severity including grade as per Platinum 5 (described below in data analysis); allied health consultations, particularly where formal PMD assessments were used by an occupational therapist or physiotherapist and any training provided; and finally, follow-up of PMD outcomes including relevant demographic characteristics for the resident.

### Data management

To maintain confidentiality, all resident data were de-identified by assigning a number prior to documentation and analysis. Approval for the study was provided by the Central Queensland University Ethics Committee, 0000022752.

### Data analysis

Descriptive statistics including frequencies and percentages were used to present the number of incidents and resulting injuries [36]. The incident severity grades specified in Platinum 5, consisted of grades one, two and three (G1-G3) and were adopted in this study. Using this classification, G1 would refer to an incident with no apparent injury, G2 for minor first aid and G3 when medical attention or transfer to hospital were required [37]. Incidents incurred during self-transfer to or from a PMD were included as a fall, as consistent with other studies [17, 18, 21], and rated as G1, G2, or G3 depending on the severity of injury. Incidents were excluded when not directly involving use of a PMD, such as a resident skin tear from staff error when using a footplate. Each incident was recorded separately and linked to the resident number, to enable patterns of occurrence to emerge. Brief simple notes to enrich the numerical data were recorded to add context to the incidents, and to group the data by content [40]. A small number of subgroup analyses, using simple frequency calculations were undertaken where appropriate. This included examining the number of incidents in the categories of resident demographic characteristics, incident severity, allied health consultations and follow-up for residents involved in PMD incidents. To project a national, annual PMD incident rate the following calculation was used. The estimated number of Australian residential aged care facilities (*n* = 2672) [41] was multiplied by the annual number of incidents documented within this study (*n* = 55) and divided by the number of facilities in the group (*n* = 33).

## Results

### Incident and PMD type

There were 55 incidents incurred by a total of 30 residents with no record of a fatality related to an incident. The findings, as presented in Table 1, show that 96% of incidents required first aid or no intervention, 67% were sustained by males and people aged over 80, and by the time of follow up 67% of the 30 residents had discontinued PMD use, or had died (not related to the PMD incident recorded).

**Table 1:** Summary of PMD use related incidents (*n* = 30 residents with 55 incidents)

**Resident #**	**AGE**	**Gender**	**Diagnosis as per ICD 11**	**Type of PMD**	**Incident Type**	**Type of Injury**	**Severity rating**	**Cognitive Ax:** PRE = before data period Post = after data period	**Allied Health Formal PMD Assessment (Ax):**	**Training:** (Doc. sessions with OT/ AHA)
#1	93	F	8, 11, 12, 13, 15	UN	C	ST	G2	Pre PAS 0 (minimal)	Nil Ax	Nil training
#2	97	F	8, 9, 10, 11, 15	UN	N	Nil	G1	Pre PAS **12.92** (moderate) Post PAS 7.74 (mild)	Nil formal Ax used	Added as training -multiple practical by OT & PT
#3	63	M	6, 12, 13, 16	PWC	C C	ST ST	G2 G2	Post PAS 2 (minimal)	Nil Ax	Nil training
# 4	85	M	5, 8, 9, 11, 15, 16	Both	C F F	ST ST ST	G2 G2 G2	PAS **16** (moderate)	Nil formal Ax used (1 × practical Ax by PT)	Nil training
#5	83	M	11, 12, 13, 15	UN	N	Nil	G1	Pre PAS 3 (minimal)	Nil formal Ax used (1 × practical Ax by PT)	Nil training
#6	84	M	5, 8, 11	UN	N	Nil	G1	Nil	MMSE 25/30 (mild)	Nil training
#7	87	M	1, 2, 5, 11, 12, 15, 22	UN	F C C C	ST ST Nil BR	G2 G2 G1 G2	Pre PAS 7 (mild)	Nil formal Ax used	Added as training -multiple practical by OT & PT
#8	76	M	6, 8, 11, 12	UN	C F F	ST ST ST	G2 G2 G2	Pre PAS 8 (mild)	Nil Ax	Nil training
#9	67	M	5, 22	UN	C	PD	G1	Pre PAS **14** (moderate)	Nil formal Ax used (1 × practical Ax by PT)	Nil training
#10	77	F	5, 6, 8, 15	UN	T	Nil	G1	PAS 4 (mild)	Nil Ax	Nil training
#11	81	F	5, 6, 8	UN	C	Nil	G1	PAS **21** (severe)	MMSE 28/30 (OT)	Nil training
#12	80	F	6, 8, 11	PWC	F T	Nil GR	G1 G2	Pre PAS 8 (mild)	PIDA 51.5% PIDA 78%	2 × OT training sessions
#13	68	M	6, 8, 11	UN	F F F M F F	Nil Nil FR Nil GR Nil	G1 G1 G3 G1 G2 G1	PAS **16.5** (severe)	Nil formal Ax used (1 × practical Ax by PT)	Nil training
#14	81	F	3, 5, 6, 8, 11, 13, 15	UN	C	BR	G2	PAS 2 (minimal)	Nil Ax	Nil training
#15	84	M	1, 5, 6, 11, 16, 21, 22	UN	C C	ST ST	G2 G2	Pre PAS 0 (minimal)	Nil Ax	Nil training
#16	86	M	6, 8, 11, 22	UN	M	Nil	G1	Nil	Nil Ax	Nil training
#17	70	F	5, 6, 8, 11, 13, 15, 22	UN	F	Nil	G1	Pre PAS 8.84 (mild)	Nil Ax	Nil training
#18	93	M	8, 9, 11, 15	PWC	C C	ST Nil	G2 G1	Pre PAS 5 (mild)	Nil Ax	Nil training
#19	75	M	5, 11, 13	PWC	C	Nil	G1	Pre PAS 4 (mild)	Nil Ax	Nil training
#20	94	M	5, 11, 12, 15, 16	UN	F	FR	G3	PAS 4 (mild)	Nil formal Ax used	Added as training -multiple practical by OT & PT
#21	72	M	5, 6, 11	PWC	T	BR	G2	Pre PAS 3 (minimal) PAS 4 (mild)	Pre SMMSE 26/30 (OT)	training in OT Ax
#22	91	M	2, 5, 8, 13, 15, 16, 22	MS	M M	Nil Nil	G1 G1	PAS 4 (mild) Post PAS 0 (minimal) Post MMSE 22/30 RN (mild) Post MMSE 23/30 Dr. (mild)	SMMSE 26/30 (OT)	Multiple OT sessions
#23	96	M	8, 11, 15	PWC	C C C	ST Nil STPD	G2 G1 G2	PAS 5.5 (mild)	Pre SMMSE 28/30 (OT)	Multiple OT sessions
#24	85	M	6, 8, 11, 13, 15	PWC	C	ST	G2	PAS 4 (mild) PAS **10** (moderate)	SMMSE 26/30 (OT)	training in OT Ax
#25	91	F	2, 5, 12, 13, 22	PWC	C C C C	ST GR Nil PD	G2 G2 G1 G1	PAS 4 (mild)	LOTCA – pass	Multiple OT sessions
#26	97	F	5, 9, 15	PWC	C	ST	G2	Pre PAS 4 (mild)	Pre SMMSE 27/30 (OT) PIDA 87.5%	Multiple AH Aid sessions
#27	71	M	1, 5, 6, 8, 11, 14, 16	PWC	C C C C	ST ST PD PD	G2 G2 G1 G1	Pre PAS 9.9 (mild)	DLOTCA-G (OT) PIDA 77.5%	Multiple OT sessions
#28	85	M	5, 6, 8, 11	PWC	C	ST	G2	Pre PAS 8 (mild) PAS **14** (moderate)	Pre SMMSE 23/30 (OT)	1 × AH Aid session
#29	74	F	2, 6, 8, 15	PWC	C	ST	G2	PAS × 5 0–5 (mild)	Nil formal Ax used	Multiple OT sessions
#30	91	M	1, 11, 12, 16, 22	PWC	C	ST	G2	PAS 3 (mild)	Pre SMMSE 30/30 (OT) PCDA 63%, PIDA 87.5%	Multiple OT sessions

KEY: (ICD-11 coding with major resident diagnoses)

01 – Infection (cellulitis, sepsis)

02 – Neoplasm (tumour)

03 - Blood (anaemia)

05 – Endocrine, nutrition, metabolic (diabetes, vitamin B12 deficiency, hypothyroid, obesity)

06 – Mental behavioural or neurodevelopmental (vascular dementia, dementia, depression, bipolar, post-traumatic stress disorder)

08 - Nervous system (cerebral palsy, Alzheimer’s disease, multiple sclerosis, hydrocephalus, Parkinson’s disease, stroke, Lewy body disease, peripheral neuropathy, epilepsy)

09 - Visual system (glaucoma, macular degeneration, legal blindness)

10 – Diseases of the Ear (hearing loss)

11 – Circulatory (hypertension, myocardial infarct, congestive cardiac failure, atrial fibrillation, ischaemic heart disease, peripheral vascular disease)

12 – Respiratory (pulmonary emboli, chronic obstructive pulmonary disease, chronic obstructive airway disease, emphysema)

13 – Digestive (cirrhosis, stomach, gastro-oesophageal reflux disease, cholecystitis, diverticulitis, oesophagitis)

14 – Skin (dermatitis, pressure injury)

15 – Musculoskeletal (osteo-arthritis, scoliosis, gout, spondylosis, frozen shoulder, osteoporosis)

16 – Genitourinary (cystitis, prostate, chronic kidney disease)

21 – Symptoms not specified elsewhere

PMD type:

UN= unclear description,

MS= motorised mobility scooter

PWC= motorised wheelchair

Both= both types owned

Severity Rating:

G1= an incident with no apparent injury

G2= mild to moderate injury is sustained, such as skin tear, bruise or graze which requires first aid.

G3= an incident with more serious injury such as a suspected fracture where medical attention or hospital transfer is indicated

Incident Type:

C= collision

F= fall

T= tipping

M= missing

N= near miss

Type of Injury:

ST= skin tear

G= graze

FR= fracture

B= bruise

Psychogeriatric Assessment Score (PAS):

0–3 = minimal impairment

4–9 = mild impairment

10–15 = moderate impairment

16–21 = severe impairment

Formal Ax (Assessment) Tools for use by Allied Health

PIDA = Powered mobility indoor driving assessment

PCDA = Powered mobility community driving assessment

MMSE = Mini mental status examination

SMMSE = Standardised mini mental status examination

LOTCA (or DLOTCA-G) = (Dynamic) Lowenstein occupational therapy cognitive assessment (for Geriatric use)

**Fig. 1:**
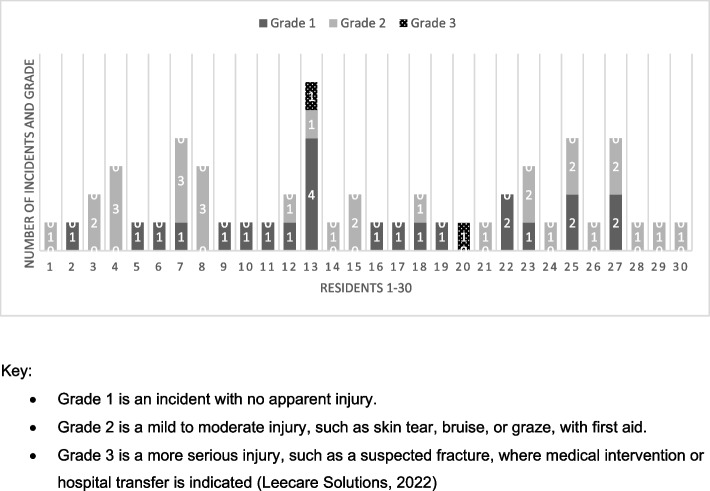
Number of incidents and injury severity grade for *N* = 30 residents

Incidents were located at 48% (*n* = 16) of the 33 facilities, with between one and four incidents at each facility. Eighteen of the residents (60%) incurred one incident only and 12 residents (40%) incurred multiple (two or more) incidents, as shown in Fig. 1. Two-thirds of all incidents were sustained by males (*n* = 20) and two-thirds by residents over the age of 80, (60’s *n* = 3), (70’s *n* = 7), (80’s *n* = 11), (90’s *n* = 9). Among the twelve residents with multiple incidents within the 12-month period, 67% were over 80 years of age and with three or more chronic conditions, as coded using ICD-11 [39]. Review of the data showed that 97% (*n* = 29) of residents had three or more chronic conditions [39] with a maximum of seven. The most prevalent codes recorded were circulatory and nervous system.

When the 55 incidents were classified by type, the majority *n* = 32 (57%) were collisions, then falls *n* = 13 (24%), followed by going missing *n* = 4 (7%), near miss *n* = 3 (6%) and tipping the PMD over *n* = 3 (6%). Half (*n* = 15) of all residents were using a device that was not defined clearly enough in the documentation to determine PMD type, 43% (*n* = 13) used a powered wheelchair, one used a mobility scooter and one resident used both, as shown in Table 1.

### Incident severity

A total of 23 incidents out of 55 (42%) were classified as G1, 30 as G2 (54%) and two (4%) as G3. Twenty-seven of the G2 incidents were breaks to the skin, with bruises making up the remaining three injuries. Skin tears to the hand, arm or leg recorded within this study were caused by collision or swipe of a table, bed, wardrobe, wall, door, tipping the wheelchair over or falling from or to the wheelchair, such as during self-transfer. The circumstances for each incident were examined, and eight incidents (15%) were found to involve another person (resident, staff, member of the public), with physical impact to the other person reported in five (9%) of these incidents. The most severe injuries recorded were two G3 incidents involving suspected fractures which required transfer to hospital, however, neither resident was moving in the PMD at the time, and both were self-transferring.

### PMD and cognitive assessments administered

Less than half of residents (40%) were recorded to have had a formal PMD assessment with results documented by an allied health professional and less than half (47%) had at least one documented training session to use a PMD. In contrast, the Psychogeriatric Assessment Scale (PAS) [38], a cognitive screening test, was performed by a registered nurse for 28 of the 30 residents (93%) as a standard requirement for completion by nurses within the organisation studied.

### Outcomes on follow-up

Follow-up notes were taken from the chart audit 9–12 months after the PMD incident and revealed that one third of residents (*n* = 10, 33%) continued to use a PMD, with four of these residents on restricted use (physical assistance was needed for safety). Among these ten residents, seven (70%) were aged in their 70’s, two in their 80’s and one in their 90’s. A circulatory diagnosis code was listed on 80% of these files and a nervous system diagnosis code on 70%, including five with stroke. All four residents on restricted use of a PMD at follow-up, had a diagnosis of stroke with hemiparesis or contracture. Another third of all residents (*n* = 11, 37%) had died within 13 months after their last incident, half of these within six months (*n* = 5). The final third of all residents (*n* = 9, 30%) had discontinued PMD use before follow-up, either by choice or request, although clear documentation on the circumstances were often lacking.

None of the seven residents with a lowered cognitive screen result (24 and below on either the Standardised Mini Mental Status Examination (SMMSE) [42] or Mini Mental Status Examination (MMSE) [43] or more than 10 on the PAS) including four with a confirmed diagnosis of dementia, continued to use a PMD at follow-up. Of the nine residents diagnosed with dementia, four had multiple incidents (44%), four died within 12 months of the incident (44%), three were no longer using the device (33%), leaving two in continuous use (22%), with one of these residents incurring multiple incidents. Among the 12 (40%) residents involved in multiple incidents, seven (58%) were no longer using the PMD at follow-up leaving five (42%) in continuous use. Only two residents with low cognitive screen results were included in the multiple incidents list, both with falls, and both had discontinued PMD use by follow-up. None of the three residents who went missing and only one with a visual impairment (25%) continued to use the device at follow-up. Table 2 displays these data.

**Table 2:** PMD Follow-up

Outcomes		Demographic information				Incident			Cognitive assessment (PAS, MMSE or SMMSE)		Allied health input (Documented)	
Continuing (*R* = Restricted use)		age	sex	Diagnoses:Number of, note		Number	Type of (majority)	Grade (highest)	Result (lowest)	Dementia diagnosis	Use of a PMD assessment tool	PMD Training
#8	**R**	70 s	M	4	(STR)	3	fall	2	mild	-	-	-
#10		70 s	F	4		1	tip	1	mild	-	-	-
#12	**R**	80 s	F	3	(STR)	2	tip	2	mild	-	Y	Y
#15		80 s	M	7		2	collision	2	minimal	-	-	-
#17	**R**	70 s	F	7	(STR)	1	fall	1	mild	-	-	-
#18		90 s	M	4	(VI)(STR)	2	collision	2	mild	-	-	-
#19		70 s	M	3		1	collision	1	mild	-	-	-
#21		70 s	M	3		1	tip	2	mild	-	Y	Y
#27	**R**	70 s	M	7	(STR)	4	collision	2	mild	Y	Y	Y
#29		70 s	F	4		1	collision	2	mild	Y	-	Y
Discontinued												
#1		90 s	F	5	(STR)	1	collision	2	minimal	-	-	-
#2		90 s	F	5	(VI)	1	near	1	**moderate**	-	-	Y
#4		80 s	M	6	(VI)(STR)	3	fall	2	**moderate**	-	-	-
#9		60 s	M	2		1	collision	1	**moderate**	-	-	-
#13		60 s	M	3	(STR)	6	missing	3	**severe**	Y	-	-
#14		80 s	F	7		1	collision	2	minimal	-	-	-
#16		80 s	M	4		1	missing	1	incomplete	-	-	-
#22		90 s	M	7		2	missing	1	mild	Y	Y	Y
#24		80 s	M	5		1	collision	2	**moderate**	Y	Y	Y
Deceased												
#3		60 s	M	4		2	collision	2	minimal	Y	-	-
#5		80 s	M	4		1	near	1	minimal	-	-	-
#6		80 s	M	3		1	near	1	**incomplete**	**Y**	Y	-
#7		80 s	M	7		4	collision	2	mild	-	-	Y
#11		80 s	F	3	(STR)	1	collision	1	**mod- sev**	Y	Y	-
#20		90 s	M	5		1	fall	3	mild	-	-	Y
#23		90 s	M	3		3	collision	2	mild	-	Y	Y
#25		90 s	F	5		4	collision	2	mild	-	Y	Y
#26		90 s	F	3	(VI)	1	collision	2	mild	-	Y	Y
#28		80 s	M	4	(STR)	1	collision	2	**moderate**	Y	Y	Y
#30		90 s	M	5		1	collision	2	mild	-	Y	Y

Key: *STR* stroke, *VI* vision impairment, *PAS* Psychogeriatric Assessment Scale (Jorm, 1995), *MMSE* Mini Mental Status Examination (Folstein, 1975), *SMMSE* Standardised Mini Mental Status Examination (Molloy, 1991)

### National, annual PMD incident rate

The number of incidents identified in this audit across 33 facilities over a 12-month period was used to project a potential annual PMD incident rate for Australian residential aged care facilities. As outlined in the data analysis section, the estimated number of 2672 Australian residential aged care facilities [41] were multiplied by the 55 annual incidents documented in this study and divided by the number of facilities studied (*n* = 33), to project 4,453 PMD incidents occurring annually across Australian residential aged care facilities.

## Discussion

This study extracted and reviewed data concerning PMD use related incidents that occur in residential aged care facilities. The data provide insights into the number of PMD use related incidents in one group of residential aged care facilities in Australia, the characteristics surrounding these incidents, and provided the means to project a national, annual incident rate for use in future research.

### Interpreting the number of PMD incidents

Aside from fatality data provided by the Australian Institute of Health and Welfare [21], this study appears to present the first examination of incident data for PMD use inside residential aged care settings. In Australian residential aged care facilities it is a requirement that every incident with injury or near miss is documented in an incident report, and this was assumed to have occurred. The number of 55 recorded PMD incidents in this study of 33 facilities was extrapolated to project the potential for 4,453 incidents to occur across Australia annually. Although this number may seem high, support is gained through comparing this figure against published community-based data, where on average 500 PMD related hospital presentations and 7 fatalities occur per year, across Australia among people aged over 60 [21]. In the community, people experiencing near misses, bruises and minor skin tears are unlikely to present to hospital, therefore, the high numbers of G1 and G2 severity grade incidents seen in residential care, would not be seen in the community data, where only G3 data are captured.

Incident reports confirmed that PMDs were in continuous use over the period of study and incidents occurred across approximately half of the facilities. Absence of PMD incidents among the other half of the facilities suggests that either there were no residents using PMDs (residents requiring higher care levels) or less likely, that documentation of incidents was incomplete. Of note was that two thirds of residents with a recorded PMD incident were no longer using their PMD one year later, as they had either discontinued, possibly due to safety concerns, or died. Furthermore, the number of residents who incurred multiple incidents was lower than single incidents, which may indicate reporting failures, or one-off incidents, or removal of the PMD after a first incident. The latter outcome is aligned with the findings of Mortenson, Miller [30] where residents described concern that their PMD would be removed if they were to have a collision. Despite a relatively small sample in this study, PMD use was shown to decline in older individuals, as evident in data showing that the continuing PMD users were predominantly from the 70’s age group. Therefore, cessation of PMD use, after incurring an incident, was demonstrated for residents from the 80’s and 90’s age groups.

### Resident demographic characteristics and patterns of incident occurrence

Australian incident research indicates that females are equally or more likely than males to be treated in hospital for PMD use related incidents, but males significantly outnumber females in fatalities [17, 18, 20]. In this study, two thirds of residents sustaining PMD related incidents were male, which may be significant given that a review of the national census data confirms that two thirds of residents in care are female [44]. Further research is required to more closely examine why males may be over-represented in incident data.

Multiple diagnoses were confirmed for almost all residents in this study and the largest proportion of recorded disorders were of the circulatory or nervous systems as consistent with frequency data from the World Health Organisation [45]. The most commonly occurring single diagnosis in this study was stroke, making up a third of the sample. Following a stroke, a combination of motor, cognitive and sensory impairments are likely to be experienced, which can prompt use of a PMD and at times impact on safety [46, 47]. Similarly, progressive disease diagnoses, and progression of a condition, were highlighted in a seminal study by Mortenson, Miller [48] which set out to determine agreement on rules for PMD use among stakeholders, to develop the first known set of overarching guidelines for PMD use in residential aged care facilities. Within the study, stakeholder groups reached consensus on support for limits being imposed when progression of a medical condition was assessed to impede safe use of a PMD [48]. Other key issues agreed to be incompatible with continuing PMD use followed a similar theme of maintaining safety, including volitional misuse such as speeding or using the device as a weapon to bump others, substance overuse, failing to learn from errors, difficulty stopping and repeated incidents [48]. The guidelines developed by Mortenson, Miller [48], described practical, measurable behaviours for assessment, however isolating the underlying impairments, particularly when cognitive or visual in origin, can also assist with the application of appropriate interventions as outlined in subsequent studies [46, 49, 50]. In the current study, cognitive and visual impairments were alluded to by nurses in the incident reports, labelled as reduced attention, information processing, reaction time, insight, memory recall, judgement, and spatial awareness. Results confirmed that more than half of the sample had mild cognitive impairment, which appears consistent with risk for having PMD use related incidents. A small number of residents had no data to indicate cognitive impairment and none of these residents had a documented allied health consultation. Review of Table 2 shows that among the group who discontinued PMD use, moderate cognitive impairment or above was prevalent and residents among the group that had died by follow-up (unrelated to their PMD incident) appeared to have received the most allied health consultation. These findings recognise the importance of cognitive skill for safe PMD use, and the increase in staff resource utilisation for assessment and training, as resident health status deteriorates.

The environment is often implicated where incidents have occurred and PMD users often look to environmental barriers as the cause [12]. In this study, the facilities were compliant with the Aged Care Quality Standards, therefore, all included basic wheelchair accessibility, with ramps, lifts, wide doorways and hallways to allow residents to move around freely [15]. This basic accessibility did not eliminate the possibility of environmental involvement in the incidents, rather it provided assurance that the facilities studied were not expected to vary significantly from other Australian facilities. The PMD incident mechanisms identified in this study were consistent with the primary mechanisms described in the literature, including collision, tipping, and falling [18, 20, 21]. However, in this study collision was the major mechanism among documented incidents, in contrast to community data, where falls significantly outnumbered collisions [21]. In addition, the community collisions documented were only those where the PMD user presented to hospital, with more than two thirds resulting in a fatality [21], whereas the collisions in residential aged care were more likely to result in a G1 or G2 (low severity) injury, which could be treated in the facility.

### The potential for a resident to sustain an injury

Despite the PMD use related incidents in this study mostly only needing first aid, the potential for more serious injury to either the PMD user or a bystander, remained present. Incidents involving collision or near miss with vehicles and furniture, wheelchairs tipping over, running over feet and residents going missing held potential for more serious injury, as shown in the data reporting hospital presentations and fatalities [16–18, 20]. The annual incident rate projected in this study, was calculated to support the development of strategies to reduce the potential for injury sustained by thousands of people each year.

The challenge of balancing autonomy with safety can be addressed by applying a theoretical framework to help understand how provision of technology can best support people to engage in activities of daily living and social participation. The Human Activity Assistive Technology (HAAT) model [51] can be used to explain the relationships between the personal characteristics of a resident (human), that enable them to mobilise and participate (activity), using a PMD (assistive technology) within a given environment (residential aged care facility). Use of this model promotes a framework for the analysis of PMD incidents with a view to building strategies to support safe PMD use in the residential aged care setting. In order to reduce the potential for serious PMD use related injury, the HAAT model [51] can be drawn upon to promote (i) the need for residential aged care staff to review the environment to ensure this supports residents to use their PMD safely, (ii) staff training to support residents to use their PMD, and (iii) resident access to expert assessment, prescription, and training from allied health staff, such as occupational therapists, when they use a PMD for the first time. PMD skills assessments and training, such as those developed for use with community-living residents by Kirby, Smith [52] and Townsend and Unsworth [53], can contribute to minimising risk of injury for users, other residents, and the associated risk of litigation or loss of income in the case of injury to staff. A future direction for this research is the development of a PMD screening tool specifically for use in residential aged care settings, that draws on findings of this study.

### Limitations

Several limitations were noted within this study. It is possible that not all incidents or near misses were reported and recorded in the data set accessed, and during data extraction some data may have been missed if not clearly linked to PMD use in the documentation. Inconsistency in terminology to separate device types (powered wheelchair or motorised mobility scooter) in incident reports, reduced clarity and limited comparison of incidents based on type, and these limitations are common across the literature.

Data to compare the number of PMD users with reported incidents, against those people who did not have incidents was not available to the research team. This was because Platinum 5 did not formally record PMD use or provide a category for PMD incidents [37]. A further database limitation encountered in this study related to the rating of incident severity. For example, an incident such as running over the foot of another person would be rated as G1 if no injury were sustained by the PMD user, regardless of injury severity sustained by the other person. Incident reports for residents remain separate, therefore, medical outcomes for an impacted resident or staff member were never associated with the PMD user’s incident record. Incident severity was therefore expected to have been significantly under-rated since injuries and outcomes sustained by other people were not included. Improved methods for recording PMD use related incidents such as provision of a category for registration of PMD use and for PMD related incidents, would be a valuable amendment to the software system used in residential care facilities. These strategies are needed to raise the profile of PMD use as a mobility option in residential aged care, and to improve tracking of PMD incidents and outcomes for more accurate analysis of data trends and future research.

## Conclusion

This study provided the first known analysis of PMD use related incidents for residents of Australian residential aged care facilities and a projected national, annual incident rate. The data collection recorded 55 PMD use related incidents for 30 residents, with no direct fatalities and two hospitalisations from incidents caused by falls during self-transfer. However, this study illuminates the potential for serious injury among residents within a projected 4,453 incidents annually, across Australia. This potential risk highlights the need for development of specific residential aged care PMD support strategies and structures including skills assessment, training for both staff and residents, and environmental audits to ensure residential facilities and local community spaces support optimal PMD use. The inclusion of a dedicated record for PMD use in residential aged care database systems is advocated so that data regarding usage, incidents involving the user and others in the facility, and associated trends can underpin future research and development of support strategies and structures to promote safe PMD use. Strategies developed must observe resident choice, and dignity of risk, striking a balance for all stakeholders in an environment where the aim is to ensure each resident lives the best life they can [15].

## Data Availability

The data that support the findings of this study are available on request from the corresponding author on reasonable request.
